# Gene Conversion and Evolution of Gene Families: An Overview 

**DOI:** 10.3390/genes1030349

**Published:** 2010-09-30

**Authors:** Tomoko Ohta

**Keywords:** interaction of gene conversion and selection, concerted evolution, generation of gene diversity

## Abstract

The importance of gene conversion for the evolution of gene families is reviewed. Four problems concerning gene conversion, *i.e.*, concerted evolution, generation of useful variation, deleterious effects, and relation to neofunctionalization, are discussed by surveying reported examples of evolving gene families. Emphasis is given toward understanding interactive effects of gene conversion and natural selection.

## 1. Introduction

Genomes of higher organisms are more dynamically evolving than previously thought, i.e., numerous duplications, deletions, translocations, etc., are frequently occurring, and provide genetic variations. Gene conversion, which is non-reciprocal transfer of genetic material, is one mechanism for such dynamics, and generates genetic variation of various kinds. Here the significance of gene conversion in evolution of gene families is discussed by surveying examples that I happen to have noticed and found interesting. It is not possible to include many significant cases in this overview, and readers will find such examples in other chapters of this special issue. 

## 2. Gene Conversion as a Mechanism of Concerted Evolution 

In genomes of higher organisms, there are various types of duplicated gene families, from those of uniform members to those of highly variable members. It seems that multigene families are well adapted to the need of organisms. In the 1970s, it had been recognized by molecular biologists that some multigene families were characterized by unusual evolutionary features such as sequence homology and related or overlapping functions among repeated members. The best studied examples were the immunoglobulin and ribosomal gene families. 

The article by Hood, Campbell and Elgin [1] was an excellent review at that time on the evolution and variation of these families. They had noted that there were various kinds of multigene families from uniform member families like the ribosomal genes to variable member families like the immunoglobulin genes, and that transfer of gene sequences to the other members belonging to the family on a chromosome was important. They argued that gene correction (gene conversion), saltarory replication, and unequal crossing over were responsible for the maintenance of sequence homology and called the process “coincidental evolution”, which is now called concerted evolution. 

**Figure 1:**
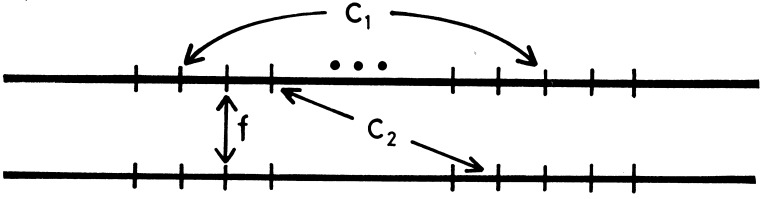
Diagram showing the meaning of the three identity coefficients for concerted evolution (from [4]).

Their argument on the immunoglobulin genes was based on the simulation study of Smith [2], which showed that by repeated unequal crossing over a single gene copy could spread and be fixed in the entire gene family. Smith’s simulation treated a single chromosomal lineage. By using the diffusion model of population genetics, the spreading of a gene copy on a chromosome by unequal crossing over was shown to be analogous to the process of mutant substitution in a finite population [3]. Next, I recognized that population genetics analyses were needed, and used a simple model of gene conversion to study the concerted evolution [4]. In the model, any gene member of a family is converted with a constant rate by another randomly chosen member belonging to the family on one chromosome. Then the gene family evolves under gene conversion, mutation and random genetic drift. In addition, inter-chromosomal (equal) crossing over is assumed to occur. For understanding the process of concerted evolution, a set of three identity coefficients are defined; f is the probability of identity of genes of allelic relationship, C1 and C2 are those of genes of non-allelic relationship on the same chromosome and on the homologous chromosome, respectively. See Figure 1 for the meaning of these identity coefficients. The process of concerted evolution may be understood by evaluating these identity coefficients, and the diversity among gene members is shown to be mainly determined by the balance among mutation, gene conversion and population size [4]. It is also shown that gene conversion and unequal crossing over may be treated similarly, if the latter process is assumed to occur in cycles of duplication and deletion with a constant gene family size [5].

It has been recognized that bias exists in allelic gene conversion, i.e., in the heterozygote, Aa, the probability of an allele A converted by an allele a is larger than that of a converted by A. This is called the biased conversion and the bias may have a significant effect on mutant dynamics even if it is very small. Walsh [6] has shown that biased gene conversion results in meiotic drive at a single locus, and is therefore equivalent to additive selection. Note that, under meiotic drive, the Aa heterozygote transmits an excess of one, and the change of allele frequency by meiotic drive can be shown to be formally equivalent to additive selection when selection is not strong. Nagylaki [7] worked out a model of biased conversion and showed that mutant spreading within a multigene family on one chromosome is equivalent to the process of spreading of an advantageous allele in a population. It is now known that biased gene conversion is fairly common, and may be important for discussions of molecular polymorphisms [8].

For concerted evolution of large gene families with uniform members, unequal crossing over is probably more important than gene conversion, because it results in simultaneous duplications or deletions of several gene copies. Eickbush and Eickbush [9] have reviewed various data on sequence divergence of ribosomal gene members on one chromosome, on homologs of a species and that between species, and argued that unequal crossing over is the major driving force of concerted evolution with sister chromatid exchange occurring more often than exchange between homologous chromosomes. 

Another problem of concerted evolution is the sequence dependent occurrence of gene conversion. Particularly the rate of occurrence of gene conversion depends upon the homology of duplicated genes. For several interesting models of this problem, see the recent review by Innan [10].

## 3. Gene Conversion Generates Useful Variations 

The best studied case of gene conversion as targets of selection is the major histocompatibility complex (MHC) of mammals. The MHC has been intensely investigated because of its importance in medicine and of the unusually high polymorphisms. Following Parham and Ohta [11], I briefly present how gene conversion may contribute to generating new functions that give pathogen resistance. Class I and Class II loci of MHC control extremely diverse immune response, and are highly polymorphic. More than 100 alleles are known in human populations at some of these loci. Balancing selection is working to increase the repertoire of immune reactions as shown by Hughes and Nei [12]. However, how are such a large number of alleles generated? Gene conversion is an efficient mechanism here. 

American Indian populations provide excellent data for studying generation of new alleles. South American Indians are known to have colonized recently. Because of the relatively small founding population, the number of alleles found in these populations is much smaller than found in other places. Also, there are differences in alleles between the South and the North Amerindians. The South Amerindian populations contain many new alleles not found in European and Asian populations, whereas the North Amerindian populations have no such alleles. Noteworthy is the fact that such new alleles have apparently been produced by recombining alleles of the founding population. Allelic and non-allelic gene conversion would produce alleles with novel functions of pathogen recognition. In fact, immunogeneticists have found many examples of two alleles that differ by a short segment of sequence that is otherwise identical to the homologous region of another allele. Non-allelic conversion seems to be much rarer than allelic conversion here, and many new alleles in South Amerindians were generated by allelic conversion. For more details, see Parham and Ohta [11]. 

The immunoglobulin gene family is another case where gene conversion contributes to producing diverse gene members. This family is known by split gene structure, and immunoglobulin diversity is generated by combinatorial use of variable region exons together with somatic mutation. In human and other mammals, members of the variable region exon cluster are quite diverse, and natural selection and gene conversion are thought to be important. In order to clarify the problem, I examined the patterns of synonymous and nonsynonymous divergence of the complementarity determining region (CDR), and of the framework region (FWR). It has been found that the difference of the divergence pattern between the CDR and the FWR is in accord with simultaneous occurrence of non-allelic gene conversion and diversity enhancing selection at CDR [13]. The logic is the following; under non-allelic gene conversion followed by natural selection, not only the number of nonsynonymous substitutions but also that of synonymous substitutions become larger in CDR than in FWR.

Other interesting cases are proteolytic enzymes and their inhibitors. Protease function is determined by a small number of amino acid residues (active sites), and protease inhibitors bind specifically to the active sites and inhibit the activities [14]. The region of binding sites of inhibitor is called the reactive center, and is characterized by hypervariability [15]. I found such hypervariability interesting and carried out statistical analyses on synonymous and nonsynonymous amino acid changes. It was found again that gene conversion followed by natural selection had been responsible for the hypervariability of the reactive center [16]. It is now known that split gene structure and alternative splicing are common in the inhibitor gene family. According to Jiang et al. [17], exon 9, which includes the reactive center, of a serin protease inhibiter (serpin) gene is duplicated, and by alternative splicing, 11 highly variable serpins are produced from the single gene in insects. The point is that exon 9 repetitive segments are highly variable, and selection operates to increase diversity together with gene conversion and unequal crossing over. Note that non-allelic gene conversion and unequal crossing over increase allelic diversity, and selection may enhance the effect. It would be difficult to say whether gene conversion or unequal crossing over is more important. According to Chen et al. [18], the ratio of gene conversion to crossing over is often larger than one in the analyses of genome-wide population genetic data. However, opposite cases where the ratio is less than one have also been reported, and it appears that it depends on the locus [18]. 

## 4. Deleterious Effects of Gene Conversion 

As with any types of mutations, gene conversion may have harmful effects, which have been observed in relation to human diseases. There are many examples of human diseases caused by gene conversion, and somatic gene conversion among duplicated genes is often responsible. Chen et al. [18] surveyed reported cases where gene conversion events, predominantly non-allelic type, cause human diseases. Their analysis suggests that about half of the donor genes of pathogenic conversion events are functional or partially functional. When the donor is a pseudogene, the accepter gene becomes nonfunctional. However, functional loss of the accepter gene is also associated with many conversion events where the donor is functional. Examples of pathogenic gene conversion are given in the table found in Chen et al. [18]. It is impressive to see various human diseases from cancer to neural tube defects are caused by gene conversion. 

Let us review two examples in some detail. One is a defect in one of the mismatch repair proteins, MSH2 and MLH1, which participate in error correction during DNA replication, and the gene defect causes genomic instability and error. According to Zhang et al. [19], defects at the MSH2 or MLH1 locus are found in patients with hereditary nonpolyposis colon carcinoma (HNPCC). For a heterozygote with a deletion at one of these loci, loss of the normal allele apparently occurs by somatic (allelic) gene conversion resulting in HNPCC. This phenomenon has been known as loss of heterozygosity in carcinogenesis. In this case, deletion via gene conversion is estimated to account for 4.3% and 10.7% of the MLH1 and MSH2 mutations, causing loss of heterozygosity, respectively [19]. Other mechanisms for the loss of heterozygosity are epigenetic modifications such as DNA methylation at the regulatory regions of these loci [20]. 

The next example is a disease caused by germ line gene conversion. Following Heinen et al. [21], the process of mutation due to gene conversion is briefly presented. Complement regulatory genes locate within the region of regulator of complement activation cluster in the human genome. Among them, genes of factor H (CFH) and five factor H related proteins (CFHL1–CFHL5) show concerted evolution. Some amino acid changing nucleotide substitutions in these genes cause atypical hemolytic uremic syndrome (aHUS). Heinen et al. [21] examined CFH mutations in 25 families of aHUS, and found that all amino acid changes involved are likely to have occurred by gene conversion between CFH and CFHL1. Particularly the de novo double amino acid changes in CFH are very difficult to explain by mutations. Identical donor sequence has been found in CFHL1, and therefore gene conversion is thought to be responsible. 

## 5. Gene Conversion vs. Neofunctionalization

Evolution of new function by gene duplication is a most interesting topic in recent years. One of the duplicated gene copies acquires new function and provides opportunities for evolution of novel systems [22]. Non-allelic gene conversion works in the opposite direction. Then what is the fate of duplicated genes under conversion and neofunctionalization? Innan [23] presented a nice two-locus model, in which selection prevents gene conversion. He has shown that the pattern of allelic and non-allelic variation is mainly determined by the balance between gene conversion and selection, and applied it to the human RHCE and RHD antigen loci. The sequence identity of the two genes is high, however, the divergence pattern of exons 1-5 is found to differ remarkably from that of exons 6-10. The Innan model predicts that the divergence between the duplicated genes is higher at the selected exons than other regions, because selection prevents accumulation of gene conversion at the selected exons. To verify this pattern at the RH loci, he classified the segregating sites among RH genes into groups; shared polymorphisms (polymorphic at both loci), and fixed different sites of the two loci. The results show that there is a cluster of fixed differences in exon 7, but that shared polymorphisms dominate in exons 1-5. The pattern is consistent with his model in which exon 7 includes the target sites of selection for new function. Innan [23] noticed that this exon includes exofacial surface, i.e., antigen reactive region, and therefore selection may be strong enough to prevent gene conversion. 

Another interesting case of conversion vs. selection is the protocadherin gene cluster. Cadherin superfamily proteins are calcium-dependent cell-adhesion molecules including cadherin and protocadherin families, which are important for tissue morphogenesis and maintenance of neuronal connection. Protocadherin is known to participate in distinguishing certain sets of neurons, and hence diversity generated by protocadherin is important. Like immunoglobulins, there are constant and variable regions. The exon containing the variable region exists in tandem clusters and diversity is generated by alternative splicing [24,25]. Gene conversion works together with selection to produce a characteristic pattern of concerted evolution of this variable region exon cluster. Noonan et al. [25] determined the structure of the protocadherin gene family of zebra fish, and examined in detail the diversity pattern of the gene family among mammals and zebra fish. By comparing the variable region exon repetitive cluster, they have found that only one particular ectodomain is homogenized, and the remaining region shows high diversity, demonstrating that this ectodomain specific sequence homogenization is a common feature of this gene family in vertebrates. In fact, trees made from the coding region of the ectodomain are characterized by very short branches as compared with trees of the remaining region. Their interpretation is that gene conversion is selected against in the variable region except for this ectodomain. Although the general picture of combinatorial use of variable region exons and homogenization of the ectodomain is conserved among species of vertebrates, Noonan et al. [25] emphasize that molecular code in development and maintenance of neuron connections are different between zebra fish and mammals, and the information content provided by this gene family is lineage specific. 

The effects of gene conversion vs. that of selection are often not as clear as the above examples. A notable case is the gene family of sex-determining genes of C. elegans. Following Rane et al. [26], the dynamic evolution of this gene family is reviewed. Sex-determining gene fog-2 and its paralog ftr-1 are important for the breeding system of C. elegans, and fog-2 participates in the hermaphroditic spermatogenesis pathway. Both protein products contain F-box domain, and divergence between the two genes shows patchwork pattern, reflecting non-allelic gene conversion. However the pattern is not as clear as the previous examples to show the counteracting effects of selection and gene conversion. Rane et al.’s analysis indicates that 1/5-1/3 of allelic sequence diversity has been generated by non-allelic gene conversion between the two loci. The possible significance of this case is the effect of gene conversion on hermaphroditism and hence on breeding systems in natural populations. 

## 6. Final Remarks

Like any biological events, gene conversion shows various effects in conjunction with other processes, giving complicated circumstances. Nevertheless, analyses of duplicated genes provide good opportunities for understanding the significance of gene conversion on functional organization of duplicated genes. Theories and examples given here are limited, however, it is impressive to see how gene conversion participates in generating diverse functions and in other processes. Also, many more interesting cases can be found in a recent review of Innan [10] and in the other chapters of this special issue, and future progress is anticipated. 

Interaction of gene conversion and natural selection is a most interesting subject for evolutionary biologists. So far, most studies on this topic are concerned with protein coding regions, because the effects may be easily recognized as compared with those at other regions. In the future, it will be interesting to extend the analyses to regulatory regions, as gene regulation is thought to be the basis of organismal development and therefore of morphological evolution. 
